# Genome-Wide Analysis of WRKY Gene Family and the Dynamic Responses of Key WRKY Genes Involved in *Ostrinia furnacalis* Attack in *Zea mays*

**DOI:** 10.3390/ijms222313045

**Published:** 2021-12-02

**Authors:** Yin Tang, Jingfei Guo, Tiantao Zhang, Shuxiong Bai, Kanglai He, Zhenying Wang

**Affiliations:** State Key Laboratory for Biology of Plant Diseases and Insect Pests, Institute of Plant Protection, Chinese Academy of Agricultural Sciences, Beijing 100193, China; tangy2324@163.com (Y.T.); guojingfei1989@126.com (J.G.); zhtiantao@163.com (T.Z.); sxbai@ippcaas.cn (S.B.); klhe@ippcaas.cn (K.H.)

**Keywords:** *Zea mays*, WRKY transcription factor, genome-wide analysis, transcriptional regulation prediction, expression profiles, *Ostrinia furnacalis*

## Abstract

WRKY transcription factors comprise one of the largest gene families and serve as key regulators of plant defenses against herbivore attack. However, studies related to the roles of WRKY genes in response to herbivory are limited in maize. In this study, a total of 128 putative maize WRKY genes (*ZmWRKYs*) were identified from the new maize genome (v4). These genes were divided into seven subgroups (groups I, IIa–e, and III) based on phylogenomic analysis, with distinct motif compositions in each subgroup. Syntenic analysis revealed that 72 (56.3%) of the genes were derived from either segmental or tandem duplication events (69 and 3, respectively), suggesting a pivotal role of segmental duplication in the expansion of the ZmWRKY family. Importantly, transcriptional regulation prediction showed that six key WRKY genes contribute to four major defense-related pathways: L-phenylalanine biosynthesis II and flavonoid, benzoxazinoid, and jasmonic acid (JA) biosynthesis. These key WRKY genes were strongly induced in commercial maize (Jingke968) infested with the Asian corn borer, *Ostrinia furnacalis*, for 0, 2, 4, 12 and 24 h in the field, and their expression levels were highly correlated with predicted target genes, suggesting that these genes have important functions in the response to *O. furnacalis*. Our results provide a comprehensive understanding of the WRKY gene family based on the new assembly of the maize genome and lay the foundation for further studies into functional characteristics of ZmWRKY genes in commercial maize defenses against *O. furnacalis* in the field.

## 1. Introduction

Plants have evolved sophisticated inducible defense mechanisms to cope with herbivore attack at different stages of growth, involving changes at the molecular, cellular, biochemical, and physiological levels [1,2,3]. These responses are generally controlled by several key genes encoding transcription activators and repressors that regulate downstream defense-related signal transduction pathways [4]. Extensive research has shown that transcription factors (TFs) are central regulators of gene expression; they play vital regulatory roles in plant defenses against herbivores [5,6,7]. TFs are promising candidates for genetic engineering due to their roles as master regulators of many defense-related genes [8]. Accordingly, deciphering the mechanistic actions of TFs is essential to future studies into insect resistance.

The WRKY TF family is among the largest TF families known in plants. Several studies have demonstrated their crucial roles in herbivore-induced plant defenses. For example, *OsWRKY53* has been reported to activate rice defenses against *Nilaparvata lugens* by activating an H_2_O_2_ burst and suppressing ethylene biosynthesis [9]. Overexpression of the *OsWRKY89* gene enhanced rice resistance to white backed planthopper [10]. In addition, *Arabidopsis AtWRKY72*, wheat *TaWRKY53*, and tomato *SIWRKY70*, have been directly involved in herbivory-induced defense responses [11,12,13]. Since the first WRKY TF, *SPF1* was cloned from sweet potato (*Ipomoea batatas* L.) [14], many WRKY genes and corresponding sequence features have been identified in various plant species. WRKY TFs are characterized by a conserved DNA-binding domain (DBD) with approximately 60 amino acids, containing the absolutely conserved signature WRKYGQK at the N-terminus, followed by a C_2_H_2_ (C-X_4-5_-C-X_22-23_-H-X-H) or C_2_HC (C-X_7_-C-X_23_-H-X-C) type zinc-finger motif at the C-terminus [15]. WRKY TFs activate and repress gene expression by recognizing and binding to W-box elements (TTGACT/C) in the promoter regions of target genes [16]. Based on the number of WRKY domains and the pattern of the zinc finger motif, WRKY TFs can be divided into three distinct groups: group I proteins, which typically contain two WRKY domains (N-terminal and C-terminal) and a C_2_H_2_-type zinc finger motif; group II proteins, which have one WRKY domain and a C_2_H_2_-type zinc finger motif, and can be further segmented into five subgroups (IIa-IIe) based on phylogeny; and group III proteins, which also have one WRKY domain, but a unique C_2_HC-type motif [17,18].

Maize (*Zea mays* L.) is the most productive and widely grown crop in the world, and an ideal model for genetic and genomic studies [19]. In recent years, several studies on genome-wide WRKY identification have been reported in succession using different versions of the maize genome. Zhang et al. identified 120 putative WRKY protein-encoding genes in the maize B73 RefGen_v3 genome [20], and Hu et al. identified 125 putative WRKY protein-encoding genes using the new high-quality maize v4 genome [21,22]. Previous studies additionally focused primarily on the roles of WRKY TFs in response to abiotic stress or during developmental processes in maize. For example, RNA-Seq expression analysis revealed that 58 ZmWRKY genes were induced in drought stress [20]. Overexpression of *ZmWRKY17* in *Arabidopsis thaliana* decreased sensitivity to ABA and may act as a negative regulator in salt stress responses through ABA signaling [23]. Similarly, overexpression of *ZmWRKY106* improved tolerance to drought and heat in transgenic *Arabidopsis* by regulating stress-related genes [24]. Although significant progress has been made in deciphering the roles of WRKY TFs in abiotic stress tolerance, fewer WRKY TF genes have been functionally characterized in biotic stress responses, particularly herbivorous attack. In this study, we performed another characterization and analysis of the WRKY gene family using the maize v4 genome. This comprehensive gene list, which revealed a few conflicts with previous studies [20,21], contributes to the understanding of functional and genetic evolution of the entire WRKY gene family in maize. We also integrated transcriptional regulation and expression analyses of WRKY genes to screen candidate key ZmWRKY genes involved in Chinese commercial maize (hybrid Jingke968) defense against the Asian corn borer, *Ostrinia furnacalis*, which is one of the most destructive pests to maize, and which causes significant yield loss in China [25]. Our results contribute to improved understanding of the ZmWRKY gene family and lay the groundwork for future research in genetic improvement of insect resistance in commercial maize.

## 2. Results

### 2.1. Identification and Characterization of WRKY Genes in Maize

A total of 128 putative WRKY genes were identified in the maize genome (B73 RefGen_v4) using a hidden Markov model (HMM) search. Conserved domain analysis confirmed that these genes contained single or double WRKY domains (based on the Pfam and SMART databases), suggesting that they are indeed WRKY gene family members. Based on their chromosomal locations, all WRKY genes identified in this study were designated sequentially as *ZmWRKY1*-*ZmWRKY128* (Table S1). Most of the genes contained the well-conserved WRKYGQK domain, although there were four variants: WRKYGEK, WKKYGQK, WRKYGGK, and WRKYRQK, which were found in six, two, seven, and one gene, respectively, distributed primarily in groups II-c and III. There was also some variation in the zinc finger motif in two of the sequences (*ZmWRKY31* and *ZmWRKY114*). Four genes (*ZmWRKY85*, *ZmWRKY108*, *ZmWRKY110*, and *ZmWRKY127*) were found to have lost the WRKYGQK domain, and one gene (*ZmWRKY106*) had lost the zinc-finger motif (Table S1).

Protein properties, including amino acid (aa) sequence length, molecular weight (MW), and isoelectric point (pI) are shown for each ZmWRKY in Table S1. In summary, the length of the encoded proteins ranged from 99 aa (*ZmWRKY7*) to 729 aa (*ZmWRKY55*), with an average of approximately 360 aa. The MW ranged from 11.22 kDa (*ZmWRKY7*) to 78.74 kDa (*ZmWRKY55*), and the pI varied from 4.55 (*ZmWRKY98*) to 10.78 (*ZmWRKY128*).

### 2.2. Classification, Phylogenetic Analysis, and Motif Composition of the ZmWRKY Genes

**Figure 1:**
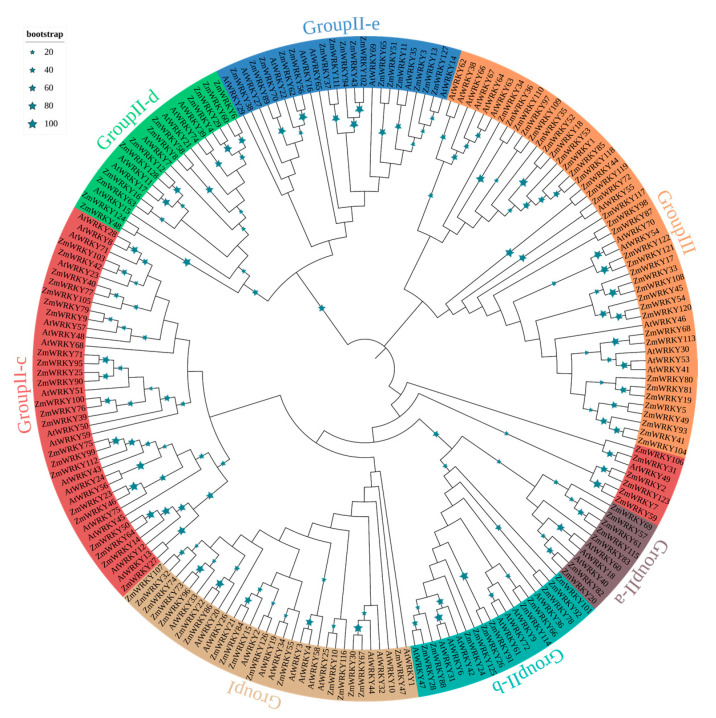
Phylogenetic analysis of WRKY gene family of *Arabidopsis* and maize. The phylogenetic tree was constructed by MEGA 7.0 using the neighbor-joining (NJ) method with 1000 bootstrap replicates. The phylogenetic tree was constructed based on the full-length protein sequences of WRKY proteins.

To better understand the evolutionary relationships of the ZmWRKY proteins, an unrooted neighbor-joining phylogenetic tree was constructed based on multiple sequence alignment of the 199 WRKY protein sequences (Table S2) from maize and *Arabidopsis* (Figure 1). Based on the classification of AtWRKYs, ZmWRKY proteins were classified into three major groups (groups I, II, and III) together with WRKYs from *Arabidopsis* (Figure 1). Among them, seventeen ZmWRKY proteins were categorized into group I. Fifteen of these contained two intact WRKY domains and C_2_H_2_-type zinc finger motifs. However, the other two members of group 1 (*ZmWRKY21* and *ZmWRKY84*) had only one complete WRKY domain each (Table S1). Seventy-five proteins were assigned to group II, and each protein in this group contained a single WRKY domain and a C_2_H_2_-type zinc finger structure. We further divided group II into five subgroups: II-a, -b, -c, -d, and -e, with 7, 11, 29, 12, and 16 members, respectively. Thirty-six ZmWRKYs with a single WRKY domain were assigned to group III because of their C_2_HC zinc-finger structure. Group II was the largest group of WRKY TFs in maize, accounting for ~57.0% of all putative ZmWRKYs (Table S1).

The phylogenetic tree was divided into two large branches. The group II subgroups aside from II-c were monophyletic (Figure 1). Groups II-a, -b, and -c were clustered together with group I, whereas groups II-d and -e were more closely related to group III (Figure 1). Phylogenetic analysis also showed that there were some closely related orthologous WRKY gene pairs between maize and *Arabidopsis* (*ZmWRKY27* and *AtWRKY13*; *ZmWRKY11* and *AtWRKY35*; *ZmWRKY127* and *AtWRKY14*; *ZmWRKY72* and *AtWRKY55*; *ZmWRKY31* and *AtWRKY49*; *ZmWRKY28* and *AtWRKY47*; *ZmWRKY10* and *AtWRKY25*; *ZmWRKY126* and *AtWRKY2*) (Figure 1).

**Figure 2:**
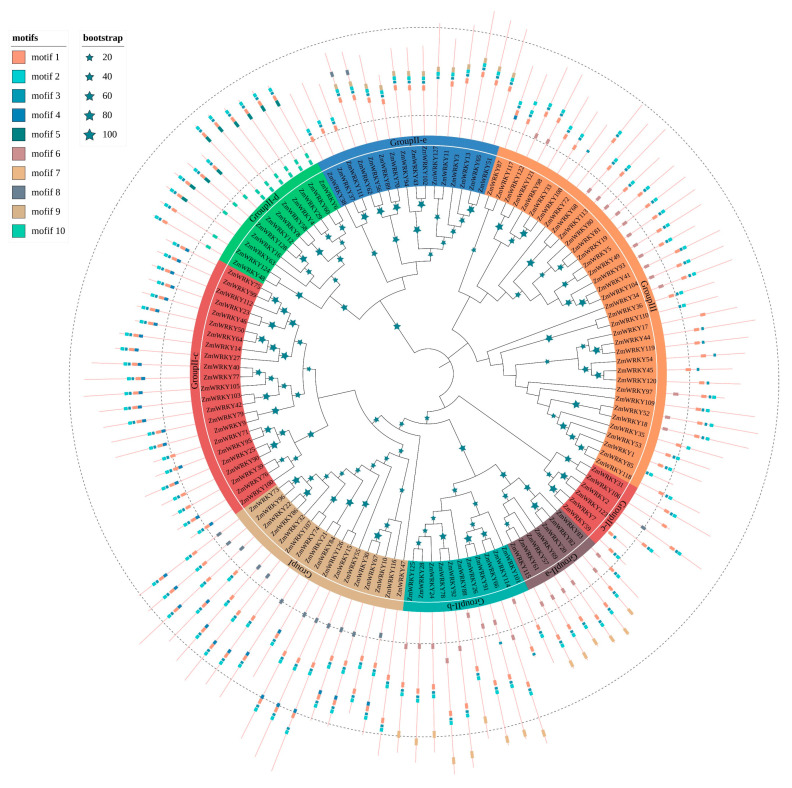
Phylogenetic relationships and motif compositions of ZmWRKY proteins. The tree was constructed using MEGA7.0 with a bootstrap of 1000 by the neighbor-joining (NJ) method. Inner layer: distribution of the conserved motifs in ZmWRKY proteins. The differently colorful boxes delineate different motifs and their positions in each ZmWRKY protein sequence.

To determine the sequence features and diversification among the ZmWRKY proteins, we predicted putative motifs using MEME [26] and identified a total of ten typical conserved motifs, which we designated as motifs 1–10. We generated a schematic distribution of these motifs among different groups based on the ZmWRKY phylogenetic relationships (Figure 2). Motifs 1, 2, and 3 were the most highly conserved and appeared most frequently in the ZmWRKY proteins. The majority of ZmWRKY proteins within the same clade generally had similar motif compositions. For example, subgroups II-a and II-b were closely related to each other, uniquely containing motifs 6 and 7. Other motifs existed only in specific groups, e.g., motifs 5 and 10 existed only in group II-d. Motif 9 was found only in group II-e, where it was present in every protein except *ZmWRKY37*, *ZmWRKY38*, and *ZmWRKY111*. Similarly, motif 4 was mainly found in groups II-c and I, and motif 8 occurred only in groups I, II-c, and II-e (Figure 2). Detailed information for each motif is shown in Table S3. Overall, the results demonstrate diversification of WRKY proteins in maize.

### 2.3. Chromosomal Location, Gene Duplication, and Collinearity Analysis of ZmWRKYs

**Figure 3:**
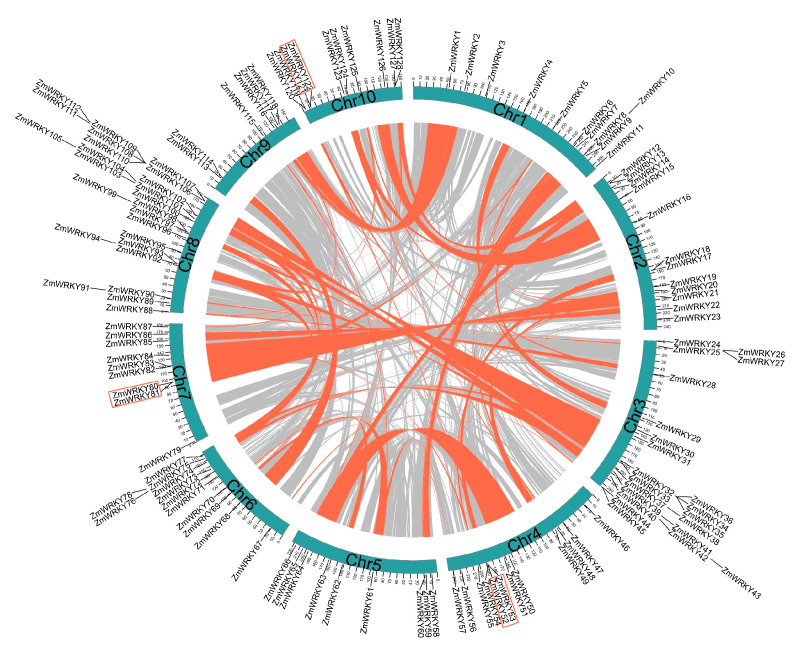
Genomic location and duplication events analysis analysis of ZmWRKY genes. Chromosomes 1–10 are represented by blue color, and the chromosome numbers are indicated at the bottom of each chromosome. Scale bar marked on the chromosome indicating chromosome lengths (Mb). Gray lines in the background indicate the synteny blocks within the maize genome. The syntenic WRKY gene pairs and tandem duplicated WRKY gene pairs are indicated with red curves and red rectangles, respectively.

To further investigate features of the ZmWRKY family, we determined the chromosomal location of each gene. All 128 putative ZmWRKY genes were unevenly distributed across the 10 chromosomes (Figure 3). Chromosome (Chr) 8 contained the highest number of ZmWRKY genes (25), followed by Chr3 (22), then Chr2, Chr4, and Chr6, (12 each). Eleven genes were located on Chr1 and 10 on Chr10. Nine ZmWRKY genes were found on both Chr5 and Chr7. In contrast, only six ZmWRKY genes were found on Chr9. The ZmWRKY genes were often located in distal telomeric regions of the chromosomes (Figure 3).

Gene duplications, including segmental duplication and tandem duplication, play a significant role in expansion of gene families [27]. Here, we identified three pairs of genes (*ZmWRKY52*/*53*, *ZmWRKY80*/*81*, and *ZmWRKY121*/*122*) derived from tandem duplication, which were located on chromosomes 4, 7, and 10, respectively (Figure 3). Notably, all three pairs of tandem duplicates were observed in group III (Figure 1) and had the same motif composition (Figure 2). Sixty-nine collinear WRKY gene pairs were also characterized (Figure 3 and Table S4), and were most often located on Chr8, followed by Chr3 then Chr6. These results indicate that segmental duplication significantly contributed to expansion of the ZmWRKY gene family.

**Figure 4:**
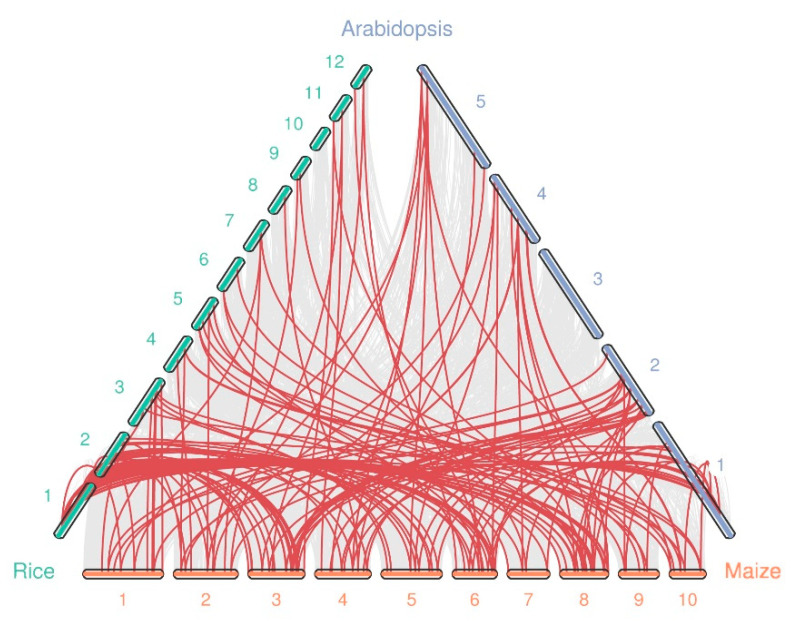
Synteny analysis of the WRKY genes between maize and two other species (rice, and *Arabidopsis*). The chromosome number is labeled at the top or bottom of each chromosome. The gray lines indicate synteny blocks in maize that are orthologous to the other genomes, while the red lines between chromosomes delineate syntenic WRKY gene pairs.

Syntenic analysis was conducted between maize and *Oryza sativa*, and *A. thaliana* to further deduce the evolutionary relationships between WRKY genes using MCScanX [28]. We found that the number of collinear gene pairs differed between species (Figure 4 and Table S5). A total of 94, and 64 orthologous gene pairs were identified between maize and rice, and *Arabidopsis*, respectively. This is likely due to the closer relationship between maize and rice than between maize and *Arabidopsis*. Additionally, we observed some gene pairs with one-for-one synteny, such as *ZmWRKY35*/*AtWRKY54* and *ZmWRKY78*/*AtWRKY59*. We speculate that these genes may have played a vital role in expansion of the WRKY gene family during evolution. In general, these results show that most orthologs exhibit unequal loss and expansion during polyploidization.

### 2.4. Expression of ZmWRKY Genes in Response to O. furnacalis Feeding

**Figure 5:**
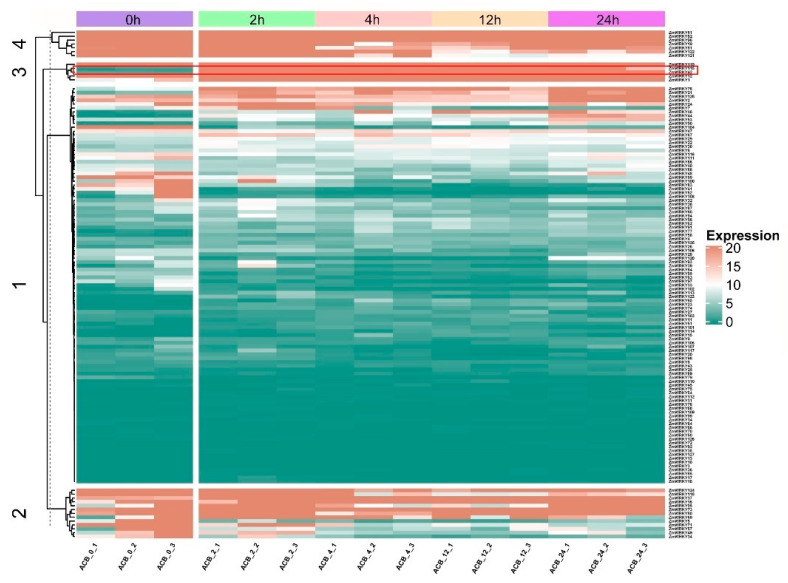
Expression profiles of the ZmWRKY genes based on transcriptome data. 0, 2, 4, 12, and 24 h represent maize leaves at 0, 2, 4, 12 and 24 h following infestation by *O. furnacalis*. Rows represent ZmWRKY gene members, while columns show different samples in the maize attacked by *O. furnacalis* at different periods of time. Red and green boxes indicated high and low expression levels of genes, respectively.

RNA-seq was carried out to elucidate the dynamic expression patterns of the 128 ZmWRKY genes in commercial maize to *O. furnacalis* attack at the mid-whorl stage (Table S6). Maize leaves were collected at 0, 2, 4, 12, and 24 h post-infestation. Not all of the predicted ZmWRKY genes were expressed under normal or herbivorous attack conditions. The 128 *ZmWRKYs* were divided into four classes by k-means clustering, based on their differential expression patterns (Figure 5). The first class contained 103 members, which showed relatively low expression in most conditions; however, expression varied at different time points after initiation of *O. furnacalis* feeding. The second and fourth classes contained thirteen and seven members, respectively, most of which were downregulated at 2, 4, 12, and 24 h post-infestation. The third class contained five members, which were strongly upregulated at all four time points post-infestation relative to 0 h. Among these, we found that *ZmWRKY42* and *ZmWRKY115* shared a similar expression pattern and clustered on the same branch. Furthermore, gene pairs in the fourth class derived from tandem duplication (*ZmWRKY80*/*81* and *ZmWRKY121*/*122*) exhibited the same expression pattern (Figure 5).

### 2.5. Prediction of Transcriptional Regulation and Differential Expression Analysis in Key ZmWRKY Genes

**Figure 6:**
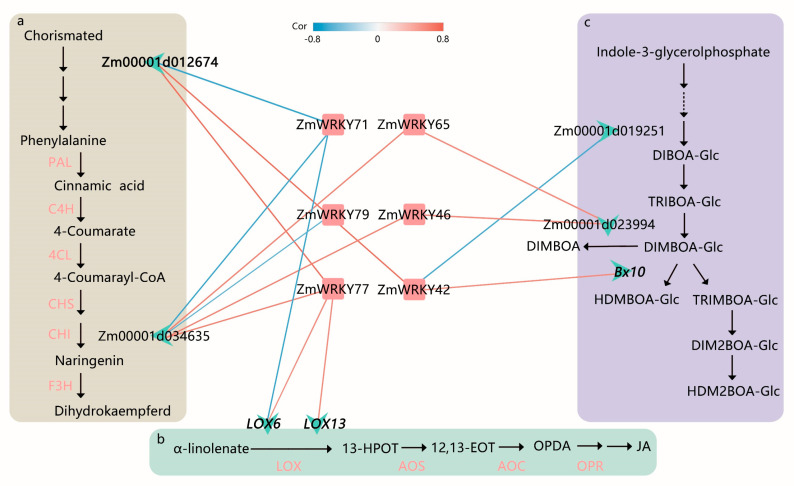
Proposed function and inferred relative position of WRKY-regulated L-phenylalanine biosynthesis II, flavonoid biosynthesis, jasmonic acid (JA) biosynthesis, and benzoxazinoid biosynthesis in maize. (**a**) Overview of L-phenylalanine biosynthesis II and flavonoid biosynthesis in maize. PAL, phenylalanine ammonia lyase; C4H, cinnamate-4-hydroxymate; 4CL, 4-coumarate: coenzyme A ligase; CHS, chalcone synthase; CHI, chalcone isomerase; F3H, flavanone-3-hydroxylase. (**b**) JA biosynthesis in maize. LOX, lipoxygenase; 13-HPOT, 13(S)-hydroperoxylinolenic acid; AOS, allene oxide synthase; 12,13-EOT, 12,13(S)-epoxylinolenic acid; AOC, allene oxide cyclase; OPDA, 12-oxocis-10,15-phytodienoic acid; OPR, 12-oxophytodienoate reductase. (**c**) Benzoxazinoid biosynthesis in maize (modified from Tzin et al. [31]). Each red square represents a WRKY gene in the network and is labeled with the gene name. Each green asterisk delegates a target gene and is labeled with the gene name or gene id, including four previously reported (in bold font). An edge color indicates correlation between WRKY TFs and target genes. The red labels indicate the target genes had been verified in the previous studies.

PlantRegMap [29] was used to infer potential regulatory interactions between ZmWRKY TFs and DEGs. Based on functional annotations from the plant metabolic network (PMN) [30], several key ZmWRKY genes and target genes were identified as being related to the flavonoid, JA signaling, and benzoxazinoid biosynthesis pathways in maize (Figure 6 and Table S7). Among them, *ZmWRKY42*, *ZmWRKY71*, and *ZmWRKY77* were predicted to bind the promoter of Zm00001d012674, which was confirmed to be involved in L-phenylalanine biosynthesis II. *ZmWRKY71*, *ZmWRKY65*, *ZmWRKY79*, *ZmWRKY46*, and *ZmWRKY77* were predicted to bind the promoter of Zm00001d034635, which is an enzyme predicted to participate in flavonoid biosynthesis. *ZmWRKY71* and *ZmWRKY77* were predicted to bind the JA pathway-related genes *LOX6* and *LOX13*. Moreover, *ZmWRKY42*, *ZmWRKY46*, and *ZmWRKY65* were predicted to bind the benzoxazinoid biosynthesis-related genes *Bx10*, Zm00001d019251, and Zm00001d023994 (Figure 6).

On this basis, we investigated the expression of these key WRKY TFs and their target genes in response to *O. furnacalis* feeding in more detail. The results showed that all key WRKY genes except for *ZmWRKY71* and *ZmWRKY79* were significantly upregulated during *O. furnacalis* feeding, and that *ZmWRKY42* was strongly induced at all four time points post-infestation (Figure S1). It should be noted that *ZmWRKY115* clustered with *ZmWRKY42*, which had a similar expression pattern and was also highly induced by *O. furnacalis* herbivory (Figure 5 and Figure S1). All predicted target genes, except for Zm00001d019251 and *LOX13*, were significantly upregulated compared with the control at all time points. Zm00001d012674 and Zm00001d023994 were found to be strongly induced by *O. furnacalis* feeding (Figure S1). In addition, the RNA-seq data showed a high correlation of these key WRKY TFs with the reported genes or candidate interacting genes (Figure 6 and Table S7).

**Figure 7:**
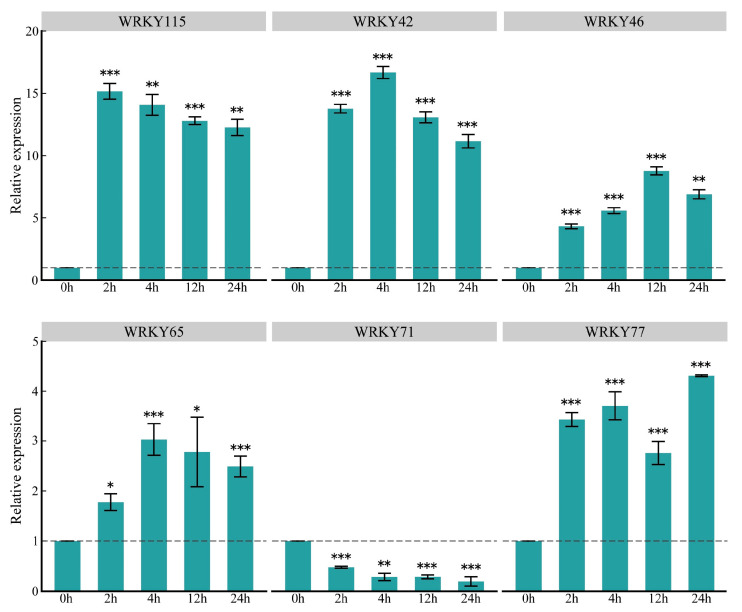
Relative expression levels of six key ZmWRKY genes induced in maize by *O. furnacalis* feeding at 2, 4, 12 and 24 h, with that of 0 h as control (set as value of 1). Values are the mean ± SD of three biological replicates. Asterisks indicate statistically significant differences in the *O. furnacalis* pre-infested maize plants compared to Control (Student’s *t*-test, ns, not significant, * *p* < 0.05, ** *p* < 0.01, *** *p* < 0.001).

To understand the expression patterns of key ZmWRKY genes at a finer scale, we quantified expression levels of these genes in response to *O. furnacalis* feeding using quantitative reverse transcription polymerase chain reaction (qRT-PCR); ZmWRKY79 was excluded due to its low expression level. The results were consistent with those from the transcriptome analysis, confirming the reliability of our RNA-seq data (Figure 7). After infestation with *O. furnacalis*, the expression levels of five of the key genes were significantly upregulated at the four time points, whereas *ZmWRKY71* exhibited the opposite expression pattern. In addition, these genes showed dynamic changes at different periods of time, indicating that the selected ZmWRKY genes were closely involved in *O. furnacalis* infestation (Figure 7).

## 3. Discussion

The WRKY TFs form a large family whose members play important roles in a variety of biological processes in plants [32,33]. Recently, a new annotated maize reference genome assembly that is more complete and accurate relative to the previous reference genome was reported [22]. This has generated new opportunities for comprehensive analysis of the WRKY gene family in maize and comparison to the WRKY families in other plants.

In this study, a total of 128 putative non-redundant WRKY genes were identified and characterized using the new maize reference genome, the number of WRKY genes is higher than that identified in the previous studies [20,21]. Compared with the previously reported by Zhang et al. [20] and Hu et al. [21], additional new ZmWRKY genes (12 and 9, respectively) were identified and renamed according to the chromosomal distribution, and some previously identified ZmWRKY genes (e.g., GRMZM2G103742) that had no corresponding gene models in maize v4 genome were considered obsolete; moreover, two genes (Zm00001d034475 and Zm00001d044315) identified by Hu et al. [21] were not found in our result based on the same maize genome (Table S1). The WRKY genes were categorized into three groups based on conserved domains and phylogenetic analysis, and the results were consistent with previous findings [21]. The molecular weights and isoelectric points of the ZmWRKY proteins showed a great deal of variation, likely due to their differing roles across a range of environments. Structural comparison of the conserved WRKY domain, which binds the promoter of target genes, presented a highly variable region responsible for new molecular activities. Motif analysis of ZmWRKY proteins demonstrated that, in general, closely related WRKY proteins from the same phylogenetic clade shared similar motif distributions and sequences, implying functional similarities. Similar results have also been reported in rice and *Arabidopsis* [34,35]. However, the sequence modifications found in some ZmWRKY proteins suggested that those family members have functionally diversified. In addition, the total number of WRKY genes identified in maize is greater than the 72 that have been identified in *Arabidopsis* [35] and the 116 in cotton [36], but fewer than the 171 identified in wheat [37] and the 188 in soybean [38] (Figure S2). This suggests a distinct degree of evolutionary expansion in the WRKY family among plant species. Wheat and soybean have undergone two whole genome duplication (WGD) events, which are responsible for the large-scale expansion of many gene families in those species [38,39]. Gene expansion can generate novel genes and broaden the function of a gene family, allowing an organism to better adapt to various environments [40]. We found that a high proportion of WRKY genes are distributed in duplicated blocks in maize, suggesting that maize WRKY genes have undergone large-scale duplication events, accelerating the expansion of the ZmWRKY gene family. In terms of evolutionary analysis, genes from maize, rice, and *Arabidopsis* exhibited extensive synteny, indicating that these WRKY genes may have existed in a common ancestor before the divergence of these lineages. Additionally, there were more syntenic blocks detected between maize and rice than between maize and *Arabidopsis*, consistent with the evolutionary relationships between monocot and eudicot species [41].

Many studies have revealed that WRKY TFs act as regulators in plant defense-related phytohormone signaling, such as the JA, salicylic acid (SA), and abscisic acid (ABA) pathways [42,43]. JA-mediated signaling is the primary activator of inducible defenses against chewing herbivore attack [44,45]. For instance, in *Nicotiana attenuate*, *NaWRKY3* and *NaWRKY6* positively control susceptibility to *Manduca sexta* by regulating JA-dependent signaling [46]. *OsWRKY70* mediates rice resistance to *Chilo suppressalis* by positively modulating JA and negatively mediating gibberellin (GA) biosynthesis [47]. Our findings may provide another case study; *ZmWRKY77* and *ZmWRKY71* were predicted to regulate lipoxygenases-related genes (*LOX6* and *LOX13*) and were involved in JA synthesis in response to *O. furnacalis* feeding. Although both were strongly induced during continuous insect attack, they showed opposite patterns of expression; *ZmWRKY77* was upregulated, whereas *ZmWRKY71* was downregulated. JA and its derivatives can induce the production of benzoxazinoids, specialized metabolites that form toxic breakdown products to deter insect feeding or inhibit digestion [48,49,50]. Here, we found that *ZmWRKY42*, *ZmWRKY46*, and *ZmWRKY65* were predicted to participate in benzoxazinoid synthesis and were highly upregulated at all tested time points post-infestation. Importantly, *ZmWRKY42* was predicted to regulate *Bx10*, which participates in methylating 2,4-dihydroxy-7-methoxy-1,4-benzoxazin-3-one glucoside (DIMBOA-Glc) to 2-hydroxy-4,7-dimethoxy-1,4-benzoxazin-3-one glucoside (HDMBOA-Glc). DIMBOA-Glc and HDMBOA-Glc are the most important benzoxazinoids in maize and have been associated with increased resistance to several lepidopteran insects, such as *Spodoptera litura*, *Mythimna separata*, and *Spodoptera frugiperda* [31,50,51,52,53].

Another crucial pathway for responding to herbivorous insect attack is the flavonoid pathway [54]. In our analysis, *ZmWRKY71*, *ZmWRKY42*, *ZmWRKY77*, *ZmWRKY46*, *ZmWRKY79*, and *ZmWRKY65* were predicted to take part in L-phenylalanine and flavonoid biosynthesis. L-phenylalanine is the precursor for a plethora of specialized metabolites involved in plant defense (e.g., flavonoids) [55]. The phylogenetic relationships between gene family members are important in functional prediction; genes clustered on the same branch may share conserved functions [56]. Previous studies have found that a TF, *ZmWRKY34* (named *ZmWRKY95* in our study), was predicted to bind the promoters of *BX6*, *BX10*, and *BX11*, and was highly induced in maize 6 h after *M. separata* feeding. It has been speculated that *ZmWRKY34* may regulate *BX6* and *BX10*/*11* at later time points [53]; however, we only found it to be significantly elevated at 2 h after *O. furnacalis* treatment. This may be due to insect species-specific differences in the time it takes the plant to selectively activate the appropriate pathways. Interestingly, *ZmWRKY95* showed a high degree of similarity with *ZmWRKY71* and *AtWRKY51*, which has been reported to mediate JA signaling and alter resistance to some virulent pathogens in *Arabidopsis* [57]. Furthermore, *AtWRKY23*, which regulates the biosynthesis of flavonol in *Arabidopsis* [58], clustered together with *ZmWRKY42*. Remarkably, *ZmWRKY115* clustered with *ZmWRKY42* in the expression profiling, suggesting a similar function for the two WRKY genes. *ZmWRKY115* was located on the same branch as *AtWRKY40*, *AtWRKY18*, and *AtWRKY60*, which are involved in the crosstalk between SA and JA signaling that affects susceptibility of *Arabidopsis* to two distinct types of pathogens [59]. *AtWRKY40* is also associated with both mechanical wounding and JA, and is consistently upregulated by *Spodoptera exigua* feeding [60]; *AtWRKY18* and *AtWRKY40* play a significant role in resistance to *S. littoralis* herbivory in *Arabidopsis* [61]. Overall, these results demonstrate that critical WRKY TFs act as positive or negative regulators of plant defense-related genes and/or signaling. Furthermore, genetic variation in different maize cultivars leads to variation in responses to herbivores attack, potentially leading to lost chemical defenses during crop domestication [49]. In this study, we identified several key candidate WRKY genes in Chinese commercially field-grown maize that is susceptible to infestation by *O. furnacalis* at the mid-whorl, which is of great practical significance in maize cultivation to enhance insect resistance in the field. Further experiments are required on transgenic plants that silence or overexpress these candidate WRKY genes to directly identify transcriptional targets of key WRKY genes, which will help to further establish their role in mediating defense responses to *O. furnacalis* in commercial maize.

## 4. Materials and Methods

### 4.1. Identification and Sequence Analysis of WRKY Genes in Maize

To comprehensively identify the WRKY genes, all maize protein sequences were downloaded from the maize genome database (B73 RefGen_v4, https://www.maizegdb.org/, accessed on 5 October 2021). The Hidden Markov Model (HMM) seed file of the WRKY domain (PF03106) was obtained from the Pfam database (http://pfam.sanger.ac.uk/, accessed on 5 October 2021) to identify maize WRKY proteins using Hmmsearch [62] with an *E*-value threshold of 0.01. Subsequently, all non-redundant maize WRKY protein sequences were validated for the presence of the WRKY domain by submitting them as search queries to the Pfam and SMART (http://smart.embl.de/, accessed on 5 October 2021) databases.

### 4.2. Sequence Alignment, Phylogenetic and Conserved Motifs Analysis

To study the phylogenetic relationships of ZmWRKY proteins and orthologs in *Arabidopsis*, sequences of AtWRKY TFs were retrieved from TAIR (http://www.arabidopsis.org, accessed on 5 October 2021). Multiple sequence alignment of the AtWRKY and ZmWRKY protein sequences was performed with the ClustalW module in MEGA 7.0 [63], and phylogenetic trees were constructed using the neighbor-joining (NJ) approach with 1000 bootstrap replicates. The phylogenetic tree was visualized using iTOL (https://itol.embl.de/, accessed on 5 October 2021) [64].

Conserved motifs of the ZmWRKY proteins were analyzed with MEME (http://alternate.meme-suite.org/tools/meme, accessed on 5 October 2021) [26]. The relevant parameters were set as follows in the analysis: number of repetitions = any; maximum number of motifs = 10; optimum width of motifs = 6 to 100. iTOL was used to visualize the phylogenetic trees and conserved motifs.

### 4.3. Chromosomal Location, Gene Duplication, and Synteny Analysis

The chromosomal distribution of ZmWRKY genes was mapped according to the physical location and length of chromosomes based on the v4 version of the maize genome annotation file (GFF3) and the corresponding genomic DNA sequences.

To explore the syntenic relationships of the WRKY genes in maize and rice, and *Arabidopsis*, whole genome protein sequences from all species were searched against themselves using BLASTP with an *E*-value threshold of 1 × 10^−10^. MCScanX [28] was used to detect the duplication types and collinear blocks using whole-genome sequences, annotation documents, and protein sequences downloaded from the Ensembl Plants database (http://plants.ensembl.org/index.html, accessed on 5 October 2021). The chromosomal distribution and inter-species syntenic analysis were visualized using Circos v0.69 [65].

### 4.4. RNA-Seq Analysis

The raw RNA-Seq data used here were generated for a previous study done in our research group [49]. We re-conducted the quality control and trimming to filter the adaptor sequences and unknown/low-quality reads with fastp [66]. Clean reads were mapped to the new maize reference genome (B73 RefGen_v4) using HISAT2 [67]. Approximately 87% of the clean reads (out of 81.87–88.93% of the total reads) were mapped to the reference genome (Table S8). Read summarization was used to obtain gene expression levels using featureCounts [68], and trimmed mean of *M*-values (TMM) [69] was used to normalize the counts. Tests for pairwise differential expression were performed in the DESeq2 R package [70], with genes having a *p* value < 0.05 and |FoldChange| > 2 considered to be differentially expressed genes (DEGs) for further analysis. Gene expression values were visualized using the ComplexHeatmap (v2.4.2) R package with expression values centralized by row. Expression patterns were clustered using k-means clustering on rows with k = 4.

### 4.5. Transcriptional Regulation Prediction and Functional Annotation

The 1.0 kb region upstream of the transcription start site for all DEGs were extracted as input sequences to infer potential regulatory interactions between ZmWRKY TFs and DEGs using PlantRegMap online (http://plantregmap.gao-lab.org/, accessed on 10 October 2021) [29]. TFs with over-represented targets in the input gene set (*p*-value ≤ 1 × 10^−5^) were identified. The Pearson correlation coefficient was calculated for enriched TFs and DEGs based on expression, and only those with a correlation coefficient greater than 0.5 and with a significant correlation (*p*-value ≤ 0.05) were submitted to the Plant Metabolic Network databases (PMN) (https://plantcyc.org/, accessed on 10 October 2021) [30] to predict the potential biological function. Finally, the filtered network was input to Cytoscape (v3.7.1) [71] to construct the regulatory network map.

### 4.6. Plant Materials and qPCR Validation

Maize genotype Jingke968 as the plant material was grown in the field; each plant was enclosed in a separate nylon cage (60 mesh). Developmentally similar and healthy maize plants were used for experiments when they were at the mid-whorl stage. Leaf samples were separately collected for extraction of total RNA and synthesis of cDNA at 0, 2, 4, 12, and 24 h after initial *O. furnacalis* infestation (20 3rd instar larvae in each plant). Three biological replicates were produced for every treatment. Detailed experimental conditions and protocols referred to Guo et al. [49].

Based on the gene expression characteristics, six candidate ZmWRKY genes were selected and their response to *O. furnacalis* infestation treatments were quantified with qRT-PCR. We performed qRT-PCR in 96-well plates using an ABI 7500 real-time PCR system (ABI, Alameda, CA, USA). Each pair of primers was tested for quality using melt curve analysis and determining their respective PCR amplification efficiency. Each assay was optimized so that the efficiency ranged between 94 and 99% (Table S9).

The total volume per reaction was 20 µL and each reaction contained 10 µL TB Green Premix Ex Taq (2×), 0.4 µL ROX Reference Dye II (50×), 2 µL cDNA, 6.8 µL ddH_2_O, and 0.4 µL each primer pair (10 µM). The reaction conditions were 95 °C for 30 s, followed by 35 cycles of 95 °C for 5 s and 60 °C for 34 s, and finally a melting curve ranging from 60 to 95 °C. Actin was used as the internal control, as described by Guo et al. [49]. Relative gene expression levels were calculated based on three biological replicates using the 2^−∆∆CT^ method [72]. To determine if there was a significant difference between the control and treatment groups, a *t*-test was employed with a threshold of 0.05.

## 5. Conclusions

In this study, a total of 128 WRKY genes were identified in an updated version of the maize genome. The genes could be divided into three main groups based on phylogenetic analyses. We conducted a systematic analysis of the genes including identification of conserved motifs, chromosomal location, gene duplication events, and synteny. Comparisons among the WRKY genes across three species (maize, rice, and *Arabidopsis*) demonstrated extensive synteny, indicating common evolutionary origins of the genes. Moreover, transcriptional regulation prediction suggested that several key WRKY genes contribute to four major defense-related pathways: L-phenylalanine biosynthesis II and flavonoid, benzoxazinoid, and JA biosynthesis. Expression of these key WRKY genes was highly correlated with expression of putative target genes, and the WRKYs were strongly induced by *O. furnacalis* feeding. This suggests that several WRKY genes have important herbivory-defensive functions in commercial maize grown in natural environments, especially in response to *O. furnacalis*. In conclusion, our results contribute to a comprehensive understanding of the ZmWRKY gene family; furthermore, we identified a set of candidate herbivory-response genes, laying the foundation for further studies in commercial maize defense against *O. furnacalis* in the field.

## Data Availability

The raw sequencing data used this study have been deposited in NCBI SRA (https://www.ncbi.nlm.nih.gov/sra, accessed on 20 October 2021) with the BioProject number PRJNA772910.

## Supplementary Materials

The following are available online at https://www.mdpi.com/article/10.3390/ijms222313045/s1.
