# Supplementary material for: Genome-Wide Analysis of WRKY Gene Family and the Dynamic Responses of Key WRKY Genes Involved in Ostrinia furnacalis Attack in Zea mays
Source: Int J Mol Sci. 2021 Dec 2;22(23):13045. doi: 10.3390/ijms222313045 (PMC8657575; doi:10.3390/ijms222313045)
Supplement: Supplementary file 1 [file ijms-22-13045-s001.zip › ijms-1475052-supplementary/Supplementary Figures.pdf]

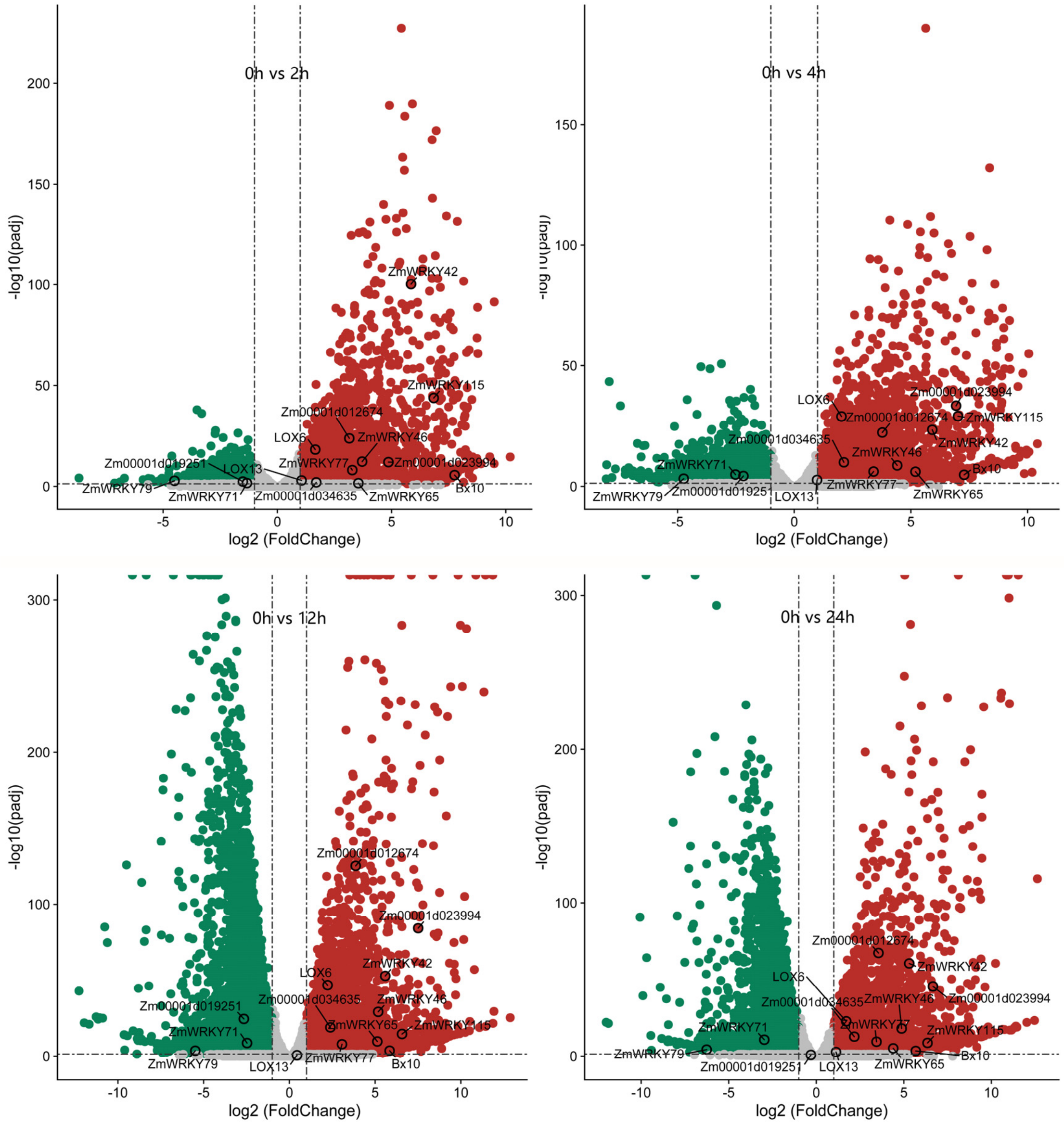

Figure S1. Volcano plots of differential expression genes (DEGs) in maize induced by *O. furnacalis* attack for 2, 4, 12 and 24 h compared with 0 h. Each gene is represented by one point on the graph. Some key ZmWRKY genes and predicted target genes are marked out in the diagram. Red and green points represent up- and down-regulated genes, respectively. Black points represent genes with no differences.

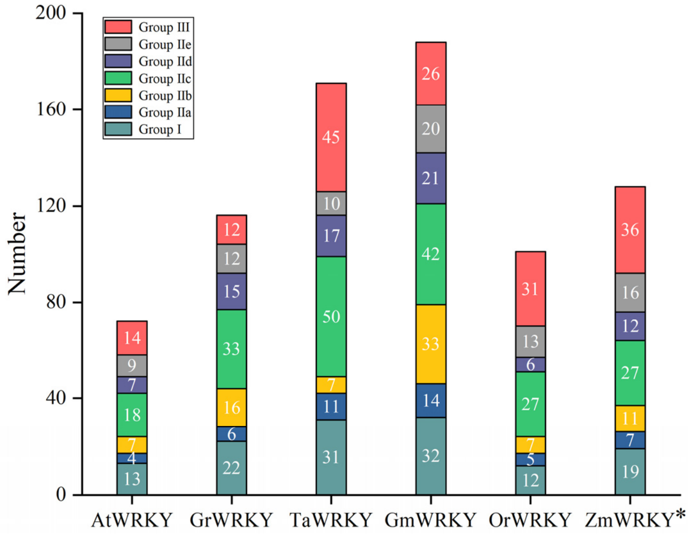

Figure S2. Summary of the number of WRKY proteins among *Arabidopsis thaliana*, *Gossypium raimondii*, *Triticum aestivum*, *Glycine max*, *Oryza rufipogon*, and *Zea mays*. * stands for in our study.
